# Novel hollow titanium dioxide nanospheres with antimicrobial activity against resistant bacteria

**DOI:** 10.3762/bjnano.10.167

**Published:** 2019-08-19

**Authors:** Carol López de Dicastillo, Cristian Patiño, María José Galotto, Yesseny Vásquez-Martínez, Claudia Torrent, Daniela Alburquenque, Alejandro Pereira, Juan Escrig

**Affiliations:** 1 Food Packaging Laboratory (Laben-Chile), Department of Science and Food Technology, Faculty of Technology, Universidad de Santiago de Chile (USACH), Obispo Umaña 050, 9170201 Santiago, Chile; 2 Center for the Development of Nanoscience and Nanotechnology (CEDENNA), 9170124 Santiago, Chile; 3 Program Center for Applied Biomedical Research, School of Medicine, Faculty of Medical Sciences, University of Santiago de Chile, 9170022 Santiago, Chile; 4 Department of Biology, Faculty of Chemistry and Biology, University of Santiago de Chile, Santiago 9170022, Chile; 5 Department of Physics, Universidad de Santiago de Chile (USACH), Av. Ecuador 3493, 9170124 Santiago, Chile; 6 Department of Sciences, Faculty of Liberal Arts, University Adolfo Ibáñez, 7941169 Santiago, Chile

**Keywords:** antimicrobial nanoparticles, atomic layer deposition, electrospinning, hollow nanospheres, titanium dioxide

## Abstract

The search for and synthesis of new antimicrobial nanostructures is important to reduce microbial incidence that induces infectious diseases and to aid in the antibiotic resistance crisis, which are two of the most pressing issues in global public health. In this work, novel, hollow, calcined titanium dioxide nanospheres (CSTiO_2_) were successfully synthesized for the first time through the combination of electrospinning and atomic layer deposition techniques. Poly(vinylpyrrolidone) (PVP) electrosprayed spherical particles were double-coated with alumina and titanium dioxide, and after a calcination process, hollow nanospheres were obtained with a radius of approximately 345 nm and shell thickness of 17 nm. The structural characterization was performed using electron microscopy, and X-ray diffraction and small-angle X-ray diffraction evidenced an anatase titanium dioxide crystalline structure. Thermogravimetric analysis and Fourier-transform infrared spectroscopy studies demonstrated the absence of polymer residue after the calcination process. The antimicrobial properties of the developed CSTiO_2_ hollow nanospheres were evaluated against different bacteria, including resistant *E. coli* and *S. aureus* strains, and when compared to commercial TiO_2_ nanoparticles, CSTiO_2_ nanospheres exhibited superior performance. In addition, the positive effect of UV irradiation on the antimicrobial activity was demonstrated.

## Introduction

Microbial contamination and the increase of multidrug bacterial resistance have become two major current concerns for food safety and human health due to the number of food-borne diseases and nosocomial infections both in developed and developing countries worldwide [1]. Thus, the search for new alternatives against microbial incidence has become an important challenge. In this context, nanoparticles (NPs) have become of great interest to researchers because of their high reactivity against both Gram-positive and Gram-negative bacteria [2]. In recent years, metal and metal oxide NPs, such as silver, gold, titanium and zinc oxide NPs, have been extensively studied due to their interesting antimicrobial character [3–5]. Titanium dioxide (TiO_2_) NPs have also attracted significant attention due to their high stability, low cost, reusability, and high photocatalytic activity [6–8]. These excellent properties have been applied in many products such as foods, catalyst support, air purification, water disinfection, antibacterial, cosmetics and solar cells [9–10]. Photocatalytic TiO_2_ favors the inactivation of microorganisms due to its strong oxidizing power by free radical generation, such as hydroxyl and superoxide anion radicals [11–12].

Metal oxide NPs have been commonly synthesized by chemical and physical methods, which can produce a high size variability, defects and agglomeration and can reduce the effectiveness of their properties [7,13–14]. The use of chemical reducing agents causes the production of larger particles with extra energy consumption and commonly low stability. Thus, the use of controlled technologies to obtain NPs with improved properties has attracted great interest [10,15]. In this work, the combination of electrospinning and atomic layer deposition (ALD) technologies are presented as an innovative strategy to develop titanium dioxide hollow nanospheres with controlled and homogeneous dimensions. Electrospinning is a technique able to produce different nanostructures, such as fibers and spherical particles, through the application of a high voltage that breaks the surface tension of the droplet of a polymeric solution located at the tip of a needle [16–17]. The morphology of the resulting nanostructures is influenced by the properties of the polymeric solution and the type of polymer. On the other hand, ALD is a novel metal oxide deposition process with excellent thickness control due to its low temperature processing and separated superficial reactions between precursor materials. Precursors are pulsed one by one over a substrate in the chamber and likewise purged to eliminate the unreacted substances and the by-product [18–20].

This is the first report on the development of metal oxide nanospheres synthesized using both electrospinning and ALD techniques. The resulting nanospheres were fully characterized by measuring the morphological, structural and thermal properties. In addition, the antimicrobial activity against common and multidrug-resistant bacteria were also studied. Crucial factors, such as the size, surface morphology and crystal structure of the NPs, were found to affect their antibacterial mechanism. Thus, the comparison of the antimicrobial activity of the developed hollow TiO_2_ nanospheres with commercial TiO_2_ NPs was also performed.

## Results and Discussion

### Material characterization

**Figure 1:**
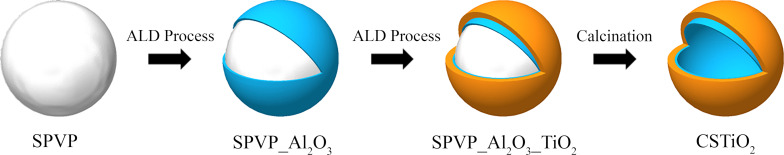
Three-step scheme to synthesize titanium dioxide nanospheres from electrosprayed SPVP spherical particles, resulting in hollow spheres after calcination, CSTiO_2_.

Titanium dioxide nanospheres were successfully obtained following the three-step procedure shown in Figure 1. First, electrosprayed spherical poly(vinylpyrrolidone) (SPVP) particles were obtained using a vertical electrospinning system. Subsequently, coated electrosprayed SPVP particles were obtained through the ALD process of Al_2_O_3_ (SPVP_Al_2_O_3_) and TiO_2_ (SPVP_Al_2_O_3__TiO_2_) layers. Prior to the deposition of titanium dioxide, a very thin deposition of Al_2_O_3_ (alumina) was necessary with the role of fixing the SPVP particles to avoid their detachment during the TiO_2_ ALD step. Finally, the calcination process, carried out at 600 °C under an air atmosphere, was applied to remove the PVP polymer from the structures, resulting in hollow titanium dioxide nanospheres (calcined TiO_2_ spheres, CSTiO_2_).

**Figure 2:**
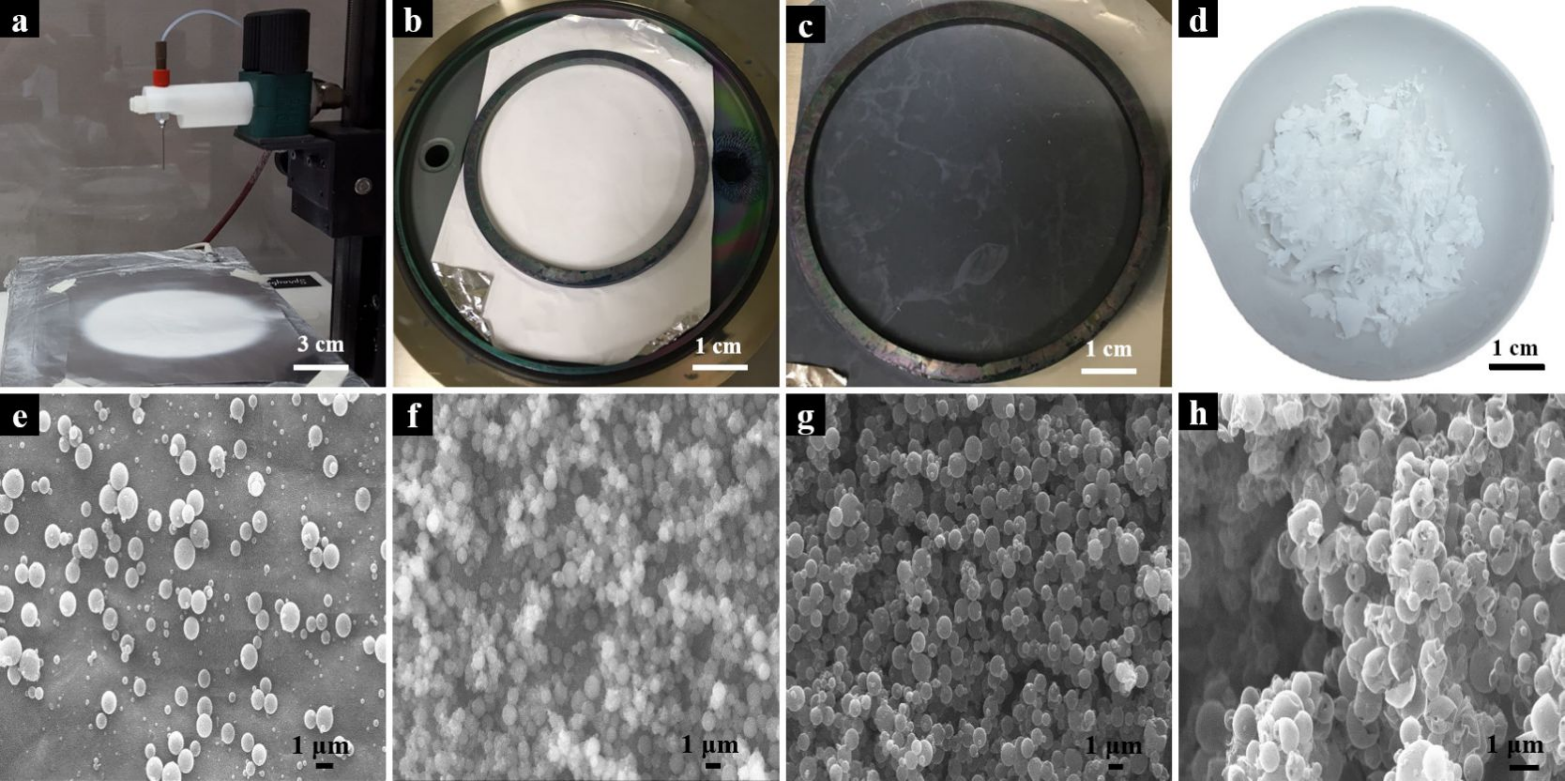
Photographs of: (a) electrosprayed spherical PVP particles (SPVP); (b) deposition of Al_2_O_3_ on SPVP by ALD (SPVP_ Al_2_O_3_); (c) deposition of TiO_2_ on the SPVP_ Al_2_O_3_ material by ALD (SPVP_ Al_2_O_3__ TiO_2_); (d) calcined samples (CSTiO_2_); Scanning electronic microscopy (SEM) images of: (e) SPVP; (f) SPVP_ Al_2_O_3_, (g) SPVP_Al_2_O_3__TiO_2_; (h) CSTiO_2_.

Figure 2 shows photographs and scanning electronic microscopy (SEM) images of structures obtained through the three-step scheme displayed in Figure 1. As Figure 2e shows, SPVP particles were successfully obtained through a stable electrospraying process by using a 20% (w/w) PVP polymeric solution. Unlike the common electrospinning process, which results in fibers by the continuous stretching of the Taylor cone through the application of a voltage to a polymeric solution with high viscosity, this case was considered an “electrospraying” process, which resulted in spherical particles due to the low-viscosity-based solution. In this process, the electric field generated monodisperse drops that contracted due to the fast evaporation of the solvent induced by Columbic explosion [21–22].

Figure 2a–c shows photographs of the three steps of the process: electrosprayed, alumina coated and alumina–titania double-coated particles. The alumina deposition maintained the initial white color of the collected electrosprayed spheres, while the TiO_2_ deposition acquired a dark bluish color. This change may be related to the number of deposition cycles that produced an optical interference due to the amount of deposited material [18,23]. SEM images presented in Figure 2e–g revealed that the morphology of the spheres was maintained after the depositions, ensuring uniformity and homogeneity.

**Figure 3:**
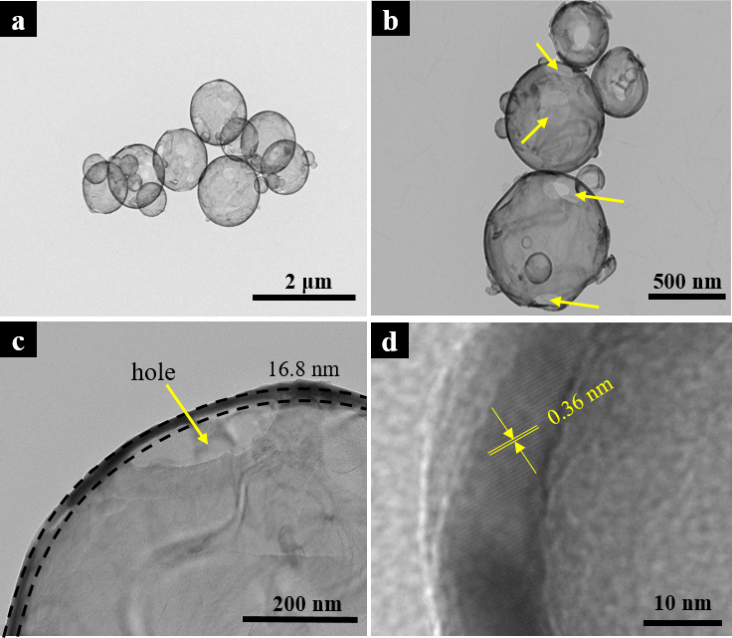
TEM images of calcined nanospheres, CSTiO_2_, at: (a) 2000×; (b) 5000×; (c) 10000× and (d) further details of the image in (c).

Subsequently, TiO_2_-coated nanospheres underwent a calcination process at 600 °C, resulting in the final, hollow nanostructures. This thermal treatment produced a change of color to white (Figure 2d), which was associated with the change of the TiO_2_ crystalline structure. Furthermore, as Figure 2h and Figure 3a–d show, SEM and transmission electronic microscopy (TEM) images confirmed that the spherical morphology was maintained after the calcination treatment.

Specifically, TEM images of CSTiO_2_ also evidenced the presence of holes in the walls of some nanostructures, which were likely produced due to the thermal degradation and release of the PVP polymeric backbone. In general, the thermal treatment at 600 °C did not generate enough pressure to break the nanosphere wall because of the double protection offered by the internal layer with Al_2_O_3_ and external layer with TiO_2_. TEM analysis also revealed that the total thickness obtained was approximately 16.8 nm after both ALD processes were performed (Figure 3c).

**Figure 4:**
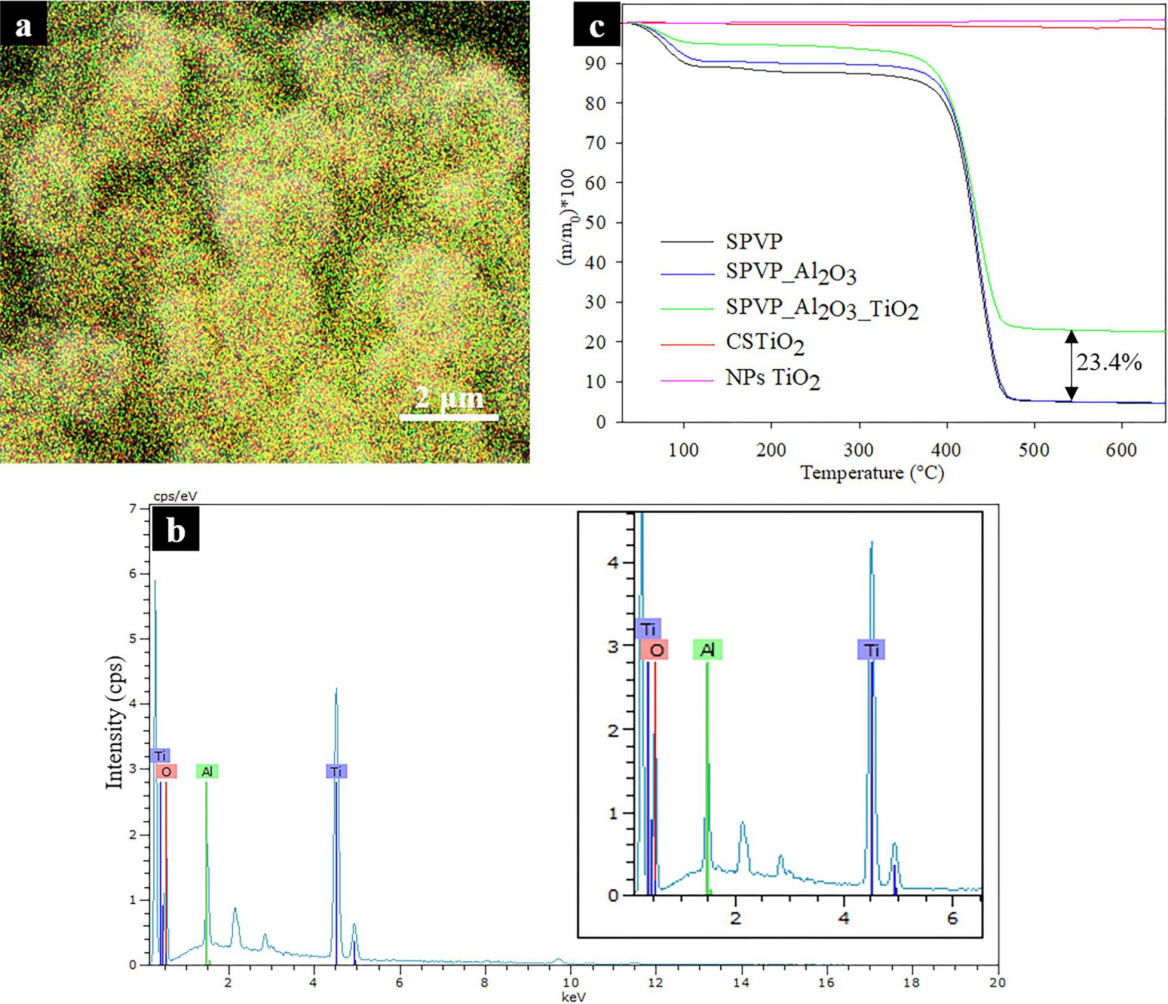
(a) EDX mapping marked with red (oxygen), green (aluminum) and yellow (titanium), (b) EDX spectrum of a CSTiO_2_ SEM image; and (c) TGA curves of the developed structures.

Energy dispersive X-ray (EDX) analysis was performed to determine the elemental composition of CSTiO_2_ and to confirm the presence of both Al_2_O_3_ and TiO_2_ layers. The EDX mapping results are shown in Figure 4a, where red, green and yellow colors correspond to oxygen, aluminum and titanium, respectively. The image did not present both layers separately, probably because the alumina layer was very thin and covered by the TiO_2_ layer. The peak corresponding to elemental titanium (Figure 4b) comprised the principal composition (38.8%) of the sample. Additional analysis regarding the chemical composition was also performed by Fourier-transform infrared spectroscopy (FTIR) in order to study the main functional groups of the sample (data found in Supporting Information File 1).

Thermogravimetric analysis (TGA) curves representing the mass loss with respect to temperature of SPVP, coated electrosprayed spheres (SPVP_Al_2_O_3_ and SPVP_Al_2_O_3__TiO_2_), and CSTiO_2_ structures are shown in Figure 4c. Except for the CSTiO_2_ sample, the uncoated and coated samples presented mass loss between 5 to 10% below 100 °C, corresponding to the dehydration process of the polymeric material in these structures. As expected, the TGA curves of structures containing PVP clearly evidenced both typical PVP degradation processes: i) the carbonic backbone decomposition around 200 °C; and ii) pyrrolidone group decomposition between 350 and 470 °C [24–26]. TGA was used to verify two important facts: i) The calcination process effectively removed the PVP polymer, since CSTiO_2_ presented the same TGA curve as the commercial TiO_2_ NPs. TGA curves of CSTiO_2_ did not show any PVP degradation process thereby demonstrating that CSTiO_2_ was PVP-free. ii) The aluminum oxide and titanium dioxide deposited during the ALD processes were (0.16 ± 0.02)% and (23.41 ± 0.47)% with respect to the polymer weight, respectively. The deposition of titania, when compared to our previous work, confirmed that the deposition onto the PVP polymer and polyvinyl alcohol presented a similar rate and it was mainly dependent on the number of cycles that determine the growth of thickness in the sample [18,27–28]. A small weigh loss of approximately 2% was found in CSTiO_2_, probably associated with the decomposition of hydroxyl groups on the titanium dioxide surface [29].

**Figure 5:**
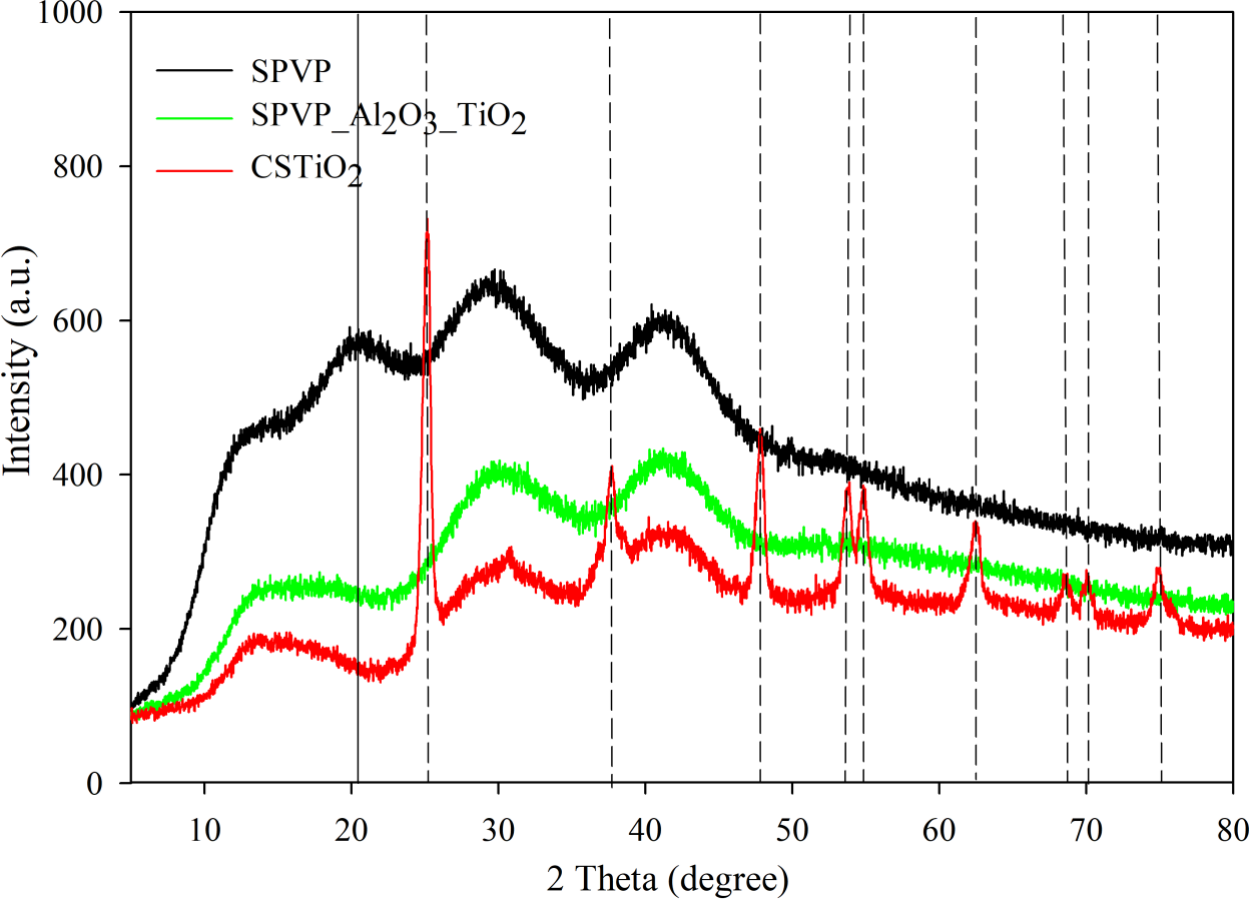
XRD diffraction patterns of: PVP electrosprayed spheres (SPVP), double coated spheres (SPVP_Al_2_O_3__TiO_2_) and hollow TiO_2_ nanospheres (CSTiO_2_).

X-ray power diffraction (XRD) analysis of the structures is shown in Figure 5. SPVP diffraction patterns showed a broad band with peak at 2θ equal to 20.3° (solid line) corresponding to the amorphous nature of the PVP polymer [30–33]. Because they were independent of the samples, the peaks at approximately 11.2, 30 and 40° corresponded to the noise baseline due to the nature of the samples. XRD diffractograms revealed that the calcination was an aggressive thermal treatment that resulted in an anatase TiO_2_ crystalline structure in the CSTiO_2_ sample [34–36]. Although previous works have mentioned that the anatase structure of TiO_2_ is a metastable structure, and can be irreversibly transformed into a stable rutile structure by heating, this process did not occur during calcination. The anatase–rutile transition occurs between 400 to 1000 °C, and it is dependent on several parameters, such as the size of the nanocrystals, impurity content, microstructure and surface area. The necessary activation energy is high and the process is slow. In addition, some works have also confirmed that the anatase phase can be stable up to 600 °C, and the rutile phase can be delayed to higher temperatures [36–38]. The anatase crystalline structure was confirmed by the presence of peaks at 25.28, 37.8, 48.05, 53.89, 55.06, 62.69, 68.76, 70.31 and 74.03º (dashed lines). This crystalline structure can present promising antimicrobial activity due to the fact that several studies have declared that this structure presents the highest antimicrobial activity owing to its higher photocatalytic nature than rutile or brookite structures [39–41].

**Figure 6:**
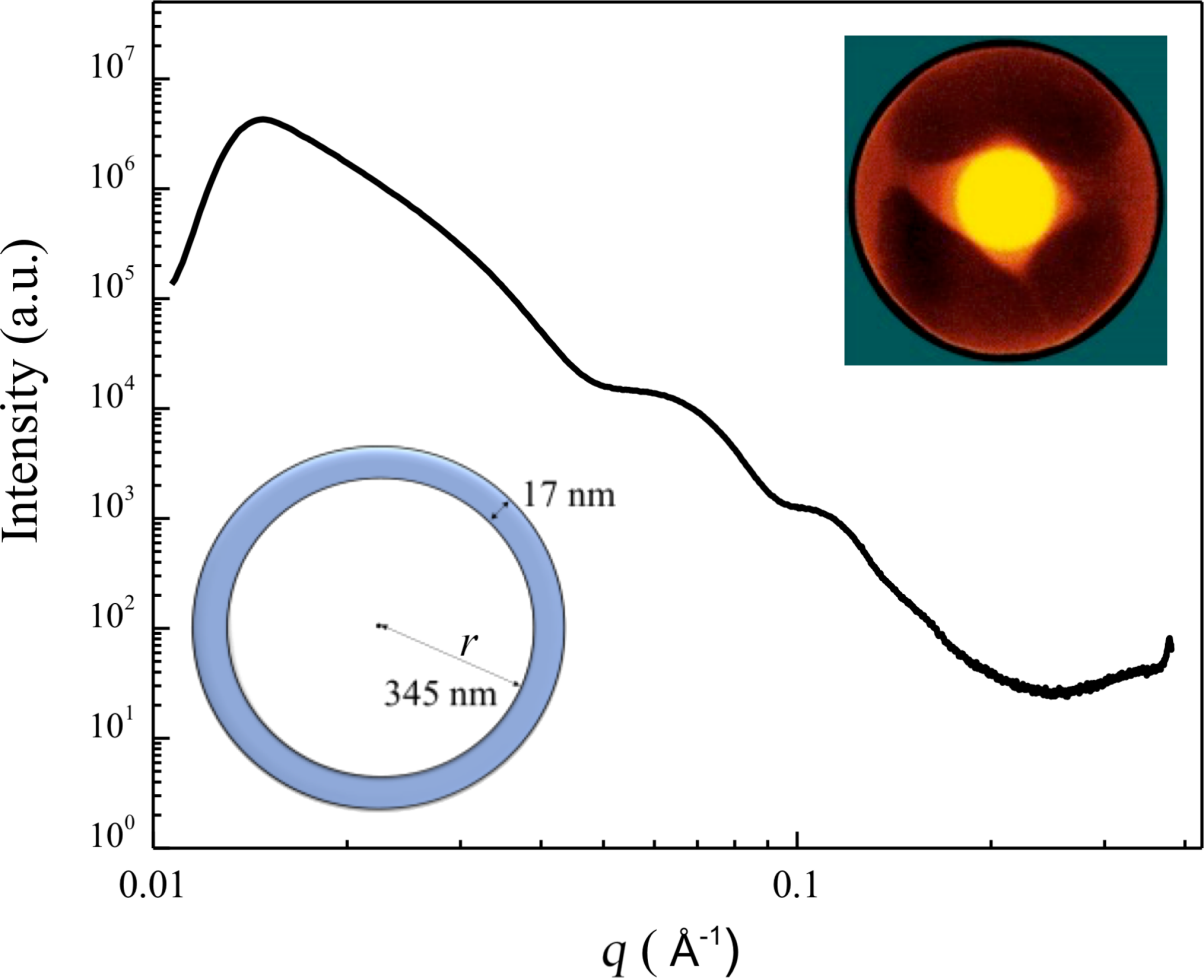
The *I*(*q*)–*q* plot of CSTiO_2_ structures obtained by SAXS.

The small-angle X-ray scattering (SAXS) technique is a powerful technique that is based on the spatial fluctuations of the electronic density of the material that allows for the measurement of polymers, alloys, and amorphous materials, whose size varies between 1 nm and several hundreds of nanometers [42]. Figure 6 shows the *I*(*q*)–*q* plot (SAXS curve) of the CSTiO_2_ structures. Additionally, the *q* in the Figure 6 is the scattering vector and *I*(*q*) is the intensity of scattering, respectively. The SAXS data were analyzed using the DIFFRAC.SAXS program that can fit and evaluate the size of the structures assuming different geometries. In this work, a core/shell particle was obtained with a particle radius of 345 nm and a shell thickness of approximately 17 nm. These results revealed an excellent agreement with the data obtained from TEM images shown in Figure 3.

### Antimicrobial activity

The improved bioactivity of nanometer-sized TiO_2_ particles is due to the area of contact and/or volume that is increased by reducing the particle size, specifically in this case the thickness (<100 nm), which allows greater interaction with molecules and proteins of the cellular membrane and a lower amount of substance [2,43–44]. In this work, the evaluation of the antibacterial activity of CSTiO_2_ with a spherical morphology and nanoscale-thickness of approximately 17 nm was evaluated and compared with traditional TiO_2_ NPs. The reduction of thickness of these nanostructures was obtained by reducing the cycles of deposition during the ALD process [18,45–46]. The antibacterial activity of CSTiO_2_ and TiO_2_ NPs was evaluated by the inhibition of growth of *Staphylococcus aureus* (control strain ATCC^®^6538TM and resistant strain MRSA 97-7 and MRSA 622-4) and *Escherichia coli* (control strain ATCC^®^25922TM and resistant strain *E. coli* 33.1). When the analysis was done using control strains, the results in Table 1 indicate that CSTiO_2_ presented an improved antibacterial activity against S. aureus and a similar activity against *E. coli* in comparison with commercial TiO_2_ NPs. Nevertheless, when assays were carried out with resistant bacteria, only CSTiO_2_ presented promising antibacterial activity against *E. coli* MRSA 33.1. This low performance could be due to the increased multidrug resistance evidenced by some bacteria due to different mechanisms, such as reduced cell permeability, target overproduction and protection [47]. As Table 1 shows, the concentration of the strongest antibiotics commonly used against these bacteria were in the range of 25–150 µg mL^−1^.

**Table 1:** Percent inhibition of nanostructures against resistant and control bacterial strains.

Antimicrobial compound	Percent inhibition of bacterial growth at 100 µg mL^−1^				
	*S. aureus* ATCC6538	*S. aureus* MRSA 97-7	*S. aureus* MRSA 622-4	*E. coli* ATCC25922	*E. coli* MRSA 33.1

CSTiO_2_	20%	0%	0%	40%	7%
TiO_2_ NPs	0%	0%	0%	35%	11%
Vancomycin (25 µg mL^−1^)	100%	100%	100%	–	–
Ampicillin (150 µg mL^−1^)	–	–	–	100%	100%

In general, CSTiO_2_ exhibited higher antibacterial capacity against Gram-negative bacteria, such as control and resistant *E. coli* strains, than Gram-positive bacteria, such as *S. aureus* ATCC 6538, MRSA 97-7 and MRSA 622-4. Different mechanisms of antimicrobial activity can be exerted by NPs. Specifically, in the case of TiO_2_ NPs, previous works have declared bactericidal activity via reactive oxygen species (ROS) generation and disruption of bacteria cell walls in the case of *E. coli*, and release and reactions of ions with thiol groups belonging to proteins of the bacterial membrane of *S. aureus* [48–49].

Probably, CSTiO_2_ presented better affinity and greater contact area with Gram-negative bacteria cells due to their cell wall structure, allowing a greater ROS formation. This tendency was already observed with TiO_2_ dioxide nanotubes developed in a previous work [18].

Due to the photocatalytic nature of titanium dioxide, different UV-A irradiation exposure times were studied in order to understand their effect on the antimicrobial activity of TiO_2_ nanostructures against *S. aureus* MRSA 97-7. The results shown in Table 2 validated that the antibacterial effect of CSTiO_2_ can be greatly increased due to the photocatalytic activity of these NPs in suspension. The antimicrobial performance of CSTiO_2_, induced by exposure to UV-A light (<390 nm), occurred generally through the generation of ROS and specifically from hydroxyl radicals (OH^•^) (through electron abstraction from water) and superoxide anion radicals O_2_^•^ (generated by the reduction of O_2_). ROS incidence can attack microbial cells through various processes, such as lipid peroxidation of cell membrane, damaging DNA and/or amino acid- and protein-based cell oxidation [50–51]. This analysis also evidenced that, although an important enhancement of CSTiO_2_ antimicrobial activity occurred within 60 min of UV irradiation, no further improvements in activity were observed with extended irradiation time. On the other hand, commercial TiO_2_ NPs did not present any antimicrobial activity against this resistant bacteria, even after a UV-irradiation time of 120 min. This fact can be due to the high resistance of this microorganism to these antimicrobial NPs at this concentration. Thus, a series of TiO_2_ NPs suspensions at increasing concentrations were examined over 60 min UV-irradiation. Assays revealed that commercial TiO_2_ NPs presented 28% reduction of *S. aureus* MRSA 97-7 strain at 300 µg mL^−1^.

**Table 2:** Effect of UV irradiation on the antimicrobial activity of nanoparticles at 100 µg mL^−1^. The percent inhibition of the *S. aureus* MRSA 97-7 strain against 100 µg/mL of CSTiO_2_ and TiO_2_ NPs as a function of irradiation time is given.

Nanoparticles	0 min irradiation	20 min irradiation	60 min irradiation	120 min irradiation

CSTiO_2_	0%	5%	37%	36%
TiO_2_ NPs	0%	0%	0%	0%

## Conclusion

This is the first report on the development of metal oxide nanospheres from the combination of electrospinning and atomic layer deposition processes. Although the purpose was the development of antimicrobial nanostructures based on titanium dioxide (due to its high photocatalytic nature), it was necessary to deposit a thin layer of alumina prior to the titania deposition to physically stabilize these low weight particles. Although previous works have demonstrated the successful deposition of metal oxides on nanofibers, the morphological change to spherical particles entailed a more difficult deposition process that was nevertheless successfully employed. Thus, hollow, spherical, antimicrobial nanostructures with a shell of 17 nm thickness were successfully obtained and antimicrobial results evidenced better performance than commercial TiO_2_ nanoparticles, principally against multidrug resistant bacteria such as the *S. aureus* strain. Antimicrobial tests also revealed that hollow TiO_2_ nanospheres present promising activity when irradiated with UV light. In addition to the antibacterial properties, these hollow nanospheres could be used for photocatalytic purposes. This represents a first work that opens up possibilities for the development of further nanostructures with different morphologies based on different metal oxides.

## Experimental

### Chemicals and microorganisms

Poly(vinylpyrrolidone) (PVP) (molecular weight 40.000), trimethylaluminium (TMA), tetrakis(dimethylamide)titanium (TDMAT) (99.99% trace metals basis) and ethanol were obtained from Sigma Aldrich (Santiago, Chile). Texas Industrial Solutions Advanced (Santiago, Chile) supplied commercial titanium dioxide nanoparticles commonly used in industrial applications, named TiO_2_ NPs.

Two clinical isolates of methicillin resistant *Staphylococcus aureus* 622-4 and 97-7, and one clinical isolate of *Escherichia coli* 33.1 were kindly donated by Dr. Marcela Wilkens from Universidad de Santiago de Chile (Chile). Control strains, *S. aureus* ATCC6538 and *E. coli* ATCC25922, were also used and provided from the same source.

A UV-A lamp bulb with 15 W from Philips (model Actinic BL TL TL-D 15W/10 1SL/25) was used.

### Development of hollow TiO_2_ nanospheres

Electrosprayed SPVP materials were obtained using a vertical electrospinning system (Spraybase^®^ power supply unit, Ireland). A 20% (w/v) PVP solution was prepared in 50% ethanol solution and stirred at room temperature until the polymer was completely dissolved. The solution was transferred to 5 mL plastic syringes and connected through a PTFE tube to a 20-gauge blunt (0.9 mm diameter) stainless steel needle charged by a high voltage power supply in the range of 0–20 kV. The parameters were previously optimized in order to obtain homogeneous spherical particles and the following values were used: flow rate 0.5 mL h^−1^, distance 12 cm and voltage 12.9 kV.

Coated electrosprayed SPVP particles were obtained through the deposition of Al_2_O_3_ and TiO_2_ layers using a Savannah S100 ALD device from Ultratech (San Jose, CA, USA) following the provider’s recipes from Cambridge NanoTech. 50 cycles of Al_2_O_3_ deposition was carried out at 80 °C using TMA and ultrapure water as precursors. Both TMA and H_2_O were unheated. The pulse times of TMA and water in the TMA/water cycle were kept at 0.015 s, while the exposure times in each half-cycle were 30 s and 60 s, respectively. Subsequently, the TiO_2_ deposition process was performed using the combination of the TDMAT precursor agent and ultrapure water for 300 cycles. These precursors were alternatively introduced into the ALD chamber at 200 °C with a pulse time of 0.1 and 0.015 s, respectively, while the exposure times were 10 s in both cases. The temperature of the TDMAT and H_2_O solutions were set at 75 °C and room temperature, respectively.

The calcination process was carried out at 600 °C for 1 h under air atmosphere.

### Characterization of structures: from SPVP to CSTiO_2_

The morphology of each structure obtained through the entire process for the development of CSTiO_2_ was observed using SEM (Zeiss EVO MA10 SEM) at 20 kV. Electrosprayed SPVP particles, before and after the ALD processes, and hollow CSTiO_2_, obtained after the polymer template removal, were studied. CSTiO_2_ particles were also observed through TEM (Hitachi HT7700 high resolution TEM) at 100 kV. Additionally, the elemental composition of CSTiO_2_ was analyzed by SEM (Vega3 Tescan SEM) with an in-column EDX detector at 15 kV.

TGA was carried out using a Mettler Toledo Gas Controller GC20 Stare System TGA/DCS. The samples (≈6 mg) were heated from 25 to 800 °C at 10 °C min^−1^ under nitrogen atmosphere (flow rate 50 mL min^−1^).

XRD patterns were measured using a Siemens diffractometer D5000 (30 mA and 40 kV) using Cu Ka (λ = 1.54 Å) radiation at room temperature. All scans were performed in a 2θ range of 2–80° at 0.02° s^−1^.

SAXS measurements were performed using a Bruker N8 Horizon SAXS system equipped with a Cu Kα radiation (λ = 1.54 Å) source and 2D VÅNTEC-500 and MONTEL optics. The data were acquired in the q-range from 0.012–0.37 Å^−1^ with a measurement time of 14.400 s in vacuum (2 mbar) at room temperature. The generator was operated at a power of 1 kW. The data processing and analysis were performed using a Bruker DIFFRAC.SAXS program.

### In vitro antibacterial activity assays using human pathogens

The antimicrobial activity of CSTiO_2_ against *E. coli* ATCC®25922TM, *E. coli* multiresistant 33.1, *S. aureus* ATCC®6538TM and methicillin-resistant *S. aureus* 97-7 and 622-4 were determined using the microdilution method established by Clinical and Laboratory Standards Institute with some modifications [52]. Briefly, stock solutions (5 mg mL^−1^) of nanoparticles in water were sonicated for 3 min and diluted in Mueller–Hinton broth (MHB) to the different two-fold assay concentrations. The obtained solution was then added to the MHB and serially two-fold diluted (in a 96-well microplate). 15 μL of inoculum at 5 × 10^6^ colony-forming units (CFUs) per mL, prepared in MHB, was then added in a final volume of 150 μL.

The effect of UV irradiation on the antimicrobial activity was investigated by irradiating the 96-well microplate with a UV-A lamp located 25 cm above the samples for 0, 20, 60, and 120 min with 150 rpm stirring at room temperature. The plates were sealed with a tight-fitting plastic cover and incubated at 37 °C for 18 h. The assays were performed in triplicate. In wells containing MHB, 15 μL of inoculum served as a negative control. The antimicrobial activity of commercial TiO_2_ NPs was also evaluated in order to compare the effectiveness of both nanostructures.

## Supporting Information

File 1 The study of structures through Fourier-transform infrared spectroscopy is described.

## References

[1] Dakal T C, Kumar A, Majumdar R S, Yadav V (2016). Front Microbiol.

[2] Wang L, Hu C, Shao L (2017). Int J Nanomed.

[3] Rai M K, Deshmukh S D, Ingle A P, Gade A K (2012). J Appl Microbiol.

[4] Kadiyala U, Turali-Emre E S, Bahng J H, Kotov N A, VanEpps J S (2018). Nanoscale.

[5] Siddiqi K S, ur Rahman A, Tajuddin, Husen A (2018). Nanoscale Res Lett.

[6] Xu Z, Zhuang C, Zou Z, Wang J, Xu X, Peng T (2017). Nano Res.

[7] Zhao Z, Zhang X, Zhang G, Liu Z, Qu D, Miao X, Feng P, Sun Z (2015). Nano Res.

[8] Feng X, Pan F, Zhao H, Deng W, Zhang P, Zhou H-C, Li Y (2018). Appl Catal, B.

[9] Qiu K, Durham P G, Anselmo A C (2018). Nano Res.

[10] Li H, Zhou Q, Gao Y, Gui X, Yang L, Du M, Shi E, Shi J, Cao A, Fang Y (2015). Nano Res.

[11] Dhanasekar M, Jenefer V, Nambiar R B, Babu S G, Selvam S P, Neppolian B, Bhat S V (2018). Mater Res Bull.

[12] De Falco G, Porta A, Del Gaudio P, Commodo M, Minutolo P, D’Anna A (2017). MRS Adv.

[13] Aytekin Aydın M T, Hoşgün H L, Dede A, Güven K (2018). Spectrochim Acta, Part A.

[14] Xu Z, Kan Y, Liu C (2018). Mater Res Bull.

[15] Pat-Espadas A M, Cervantes F J (2018). J Chem Technol Biotechnol.

[16] Liu D, Zhang C, Dong G, Xu C, Liu D, Lv Y, Zhong B, Wang B (2018). Mater Res Express.

[17] Liu X, Yang Y, Yu D-G, Zhu M-J, Zhao M, Williams G R (2019). Chem Eng J.

[18] López de Dicastillo C, Patiño C, Galotto M, Palma J, Alburquenque D, Escrig J (2018). Nanomaterials.

[19] Porro S, Bejtka K, Jasmin A, Fontana M, Milano G, Chiolerio A, Pirri C F, Ricciardi C (2018). Nanotechnology.

[20] Ricci P C, Laidani N, Chiriu D, Salis M, Carbonaro C M, Corpino R (2018). Appl Surf Sci.

[21] Cui L, Liu Z-P, Yu D-G, Zhang S-P, Bligh S W A, Zhao N (2014). Colloid Polym Sci.

[22] Munir M M, Suryamas A B, Iskandar F, Okuyama K (2009). Polymer.

[23] Edy R, Zhao Y, Huang G S, Shi J J, Zhang J, Solovev A A, Mei Y (2016). Prog Nat Sci: Mater Int.

[24] Mallakpour S, Behranvand V (2015). Prog Org Coat.

[25] Mallakpour S, Jarang N (2018). Polym Bull.

[26] Hulsey S, Absar S, Sultana Q N, Sabet S M, Mahfuz H, Khan M (2018). Polym Compos.

[27] Kavre Piltaver I, Peter R, Šarić I, Salamon K, Jelovica Badovinac I, Koshmak K, Nannarone S, Delač Marion I, Petravić M (2017). Appl Surf Sci.

[28] Bishal A K, Sukotjo C, Takoudis C G (2017). J Vac Sci Technol, A.

[29] Xiao Z, Guo P, Sun N (2017). J Appl Polym Sci.

[30] Mangalam R, Thamilselvan M, Selvasekarapandian S, Jayakumar S, Manjuladevi R (2017). Ionics.

[31] Saroj A L, Singh R K, Chandra S (2013). Mater Sci Eng, B.

[32] Hong H-K, Park C-K, Gong M-S (2010). Bull Korean Chem Soc.

[33] Bai J, Li Y, Sun L, Zhang C, Yang Q (2009). Bull Mater Sci.

[34] López de Dicastillo C, Roa K, Garrido L, Pereira A, Galotto M (2017). Polymers (Basel, Switz).

[35] Kuchi C, Harish G S, Reddy P S (2018). Ceram Int.

[36] Subhapriya S, Gomathipriya P (2018). Microb Pathog.

[37] Wang Z, Saxena S K, Pischedda V, Liermann H P, Zha C S (2001). J Phys: Condens Matter.

[38] Mendoza-Anaya D, Salas P, Angeles-Chávez C, Pérez-Hernández R, Castaño V M (2004). Rev Mex Fis.

[39] Yoganarasimhan S R, Rao C N R (1962). Trans Faraday Soc.

[40] Nagalakshmi M, Karthikeyan C, Anusuya N, Brundha C, Basu M J, Karuppuchamy S (2017). J Mater Sci: Mater Electron.

[41] Purbia R, Borah R, Paria S (2017). Inorg Chem.

[42] Shi W M, Dai X J, Yang G C, Xu Y, Liu Y (2013). Mater Sci Forum.

[43] Esfandiari N, Simchi A, Bagheri R (2014). J Biomed Mater Res, Part A.

[44] Cushing B L, Kolesnichenko V L, O'Connor C J (2004). Chem Rev.

[45] Pessoa R S, dos Santos V P, Cardoso S B, Doria A C O C, Figueira F R, Rodrigues B V M, Testoni G E, Fraga M A, Marciano F R, Lobo A O (2017). Appl Surf Sci.

[46] Park J Y, Choi S-W, Kim S S (2010). Nanotechnology.

[47] Baptista P V, McCusker M P, Carvalho A, Ferreira D A, Mohan N M, Martins M, Fernandes A R (2018). Front Microbiol.

[48] Li Y, Zhang W, Niu J, Chen Y (2012). ACS Nano.

[49] Roy A S, Parveen A, Koppalkar A R, Ambika Prasad M V N (2010). J Biomater Nanobiotechnol.

[50] Brunet L, Lyon D Y, Hotze E M, Alvarez P J J, Wiesner M R (2009). Environ Sci Technol.

[51] Fu G, Vary P S, Lin C-T (2005). J Phys Chem B.

[52] Barry A L, Craig W A, Nadler H (1999). Methods for Determining Bactericidal Activity of Antimicrobial Agents.

