# Supplementary material for: Novel hollow titanium dioxide nanospheres with antimicrobial activity against resistant bacteria
Source: Beilstein J Nanotechnol. 2019 Aug 19;10:1716–25. doi: 10.3762/bjnano.10.167 (PMC6720579; doi:10.3762/bjnano.10.167)
Supplement: File 1 — The study of structures through Fourier-transform infrared spectroscopy is described. [file Beilstein_J_Nanotechnol-10-1716-s001.pdf]

BEILSTEIN JOURNAL OF NANOTECHNOLOGY

Supporting Information

for

Novel hollow titanium dioxide nanospheres with antimicrobial activity against resistant bacteria

Carol López de Dicastillo, Cristian Patiño, María José Galotto, Yesseny Vásquez-Martínez, Claudia Torrent, Daniela Alburquenque, Alejandro Pereira and Juan Escrig

Beilstein J. Nanotechnol. **2019,** *10,* 1716–1725. doi:10.3762/bjnano.10.167

The study of structures through Fourier-transform infrared spectroscopy is described

License and Terms: This is a supporting information file under the terms of the Creative Commons Attribution License (http://creativecommons.org/licenses/by/4.0). Please note that the reuse, redistribution and reproduction in particular requires that the authors and source are credited.
The license is subject to the *Beilstein Journal of Nanotechnology* terms and conditions: (https://www.beilstein-journals.org/bjnano)

ESM.1. Fourier transform infrared spectroscopy (FTIR)

The presence of specific functional groups in the developed materials was analyzed by spectrometer equipment Brucker Alpha with accessory to make transmission spectra. Pellets with sample and potassium bromide (KBr) were prepared by pressure, and the spectra were obtained in a range from 4000 to 400 cm^{-1}, with a resolution of 2 cm^{-1} and 64 scans.

As seen in Figure S1, excepting $CSTiO_2$, all spectra showed characteristic bands of bending and stretching vibrations of the main functional groups of PVP polymer, and the positions of the adsorption bands and their assignments of the samples are presented in Table S1. Comparing to literature, peaks presented slight displacements possibly due to the electrospinning process that commonly decrease the degree of crystallinity of polymers due to fast evaporation (already seen on amorphous structure in DRX diffractograms) and the presence of residual ethanol from the process.

Calcined titanium dioxide nanospheres did not show any characteristic PVP peaks, confirming calcination process completely eliminated the polymer. Small peaks at 3436, 1641, 800 and 420 cm^{-1} were observed corresponding to the interaction between the OH groups, the flexion of Ti–OH bonds and the presence of Ti–O bonds characteristic of the anatase structure, respectively. The broad band appeared between 420 and 800 cm^{-1} is related to the stretching vibrations of Ti–O, Ti-O–C and Ti–O–Ti bonds. Other studies have already registered a similar broad band due to the vibration of Ti–O–Ti bonds in TiO_2 lattice.

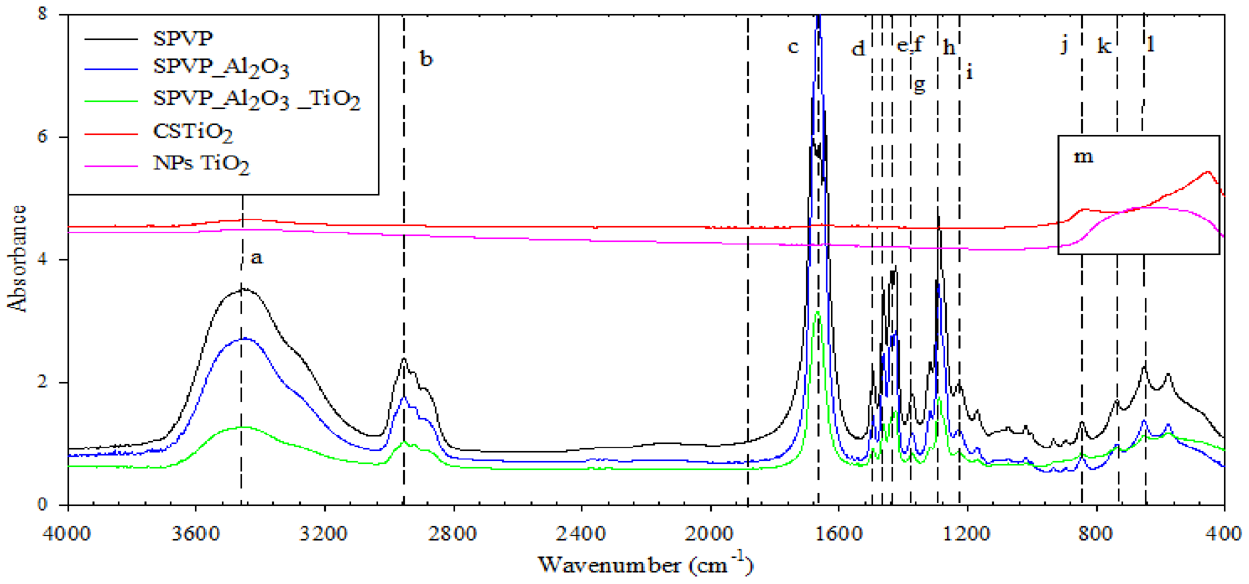

Figure S1: FTIR spectra of all developed structures and TiO_2 NPs.

Table S1: Characteristic wavenumbers (cm^{-1}) associated to assignments of FTIR absorption bands of nanostructures tagged in Figure S1.

**Peaks**	**PVP**	**$CSTiO_2$**	**Assignment**
a	3450	–	OH stretching
b	2950	–	CH_2 asymmetric stretching
c	1652	–	C=O stretching
d	1495	–	Vibration of C=N (pyridine ring)
e	1463	–	C-H bending of CH_2
f	1439	–	CH_2 wagging
g	1373	–	Swinging vibration of C–H in CH_2 group
h	1289	–	N–OH stretching
i	1230	–	C–C stretching out of plane ring
j	846	–	CH_2 twisting
k	739	–	CH_2 bending
l	644	–	C–N bending
m	–	400-850	Ti–O–Ti bonding
